# Legged Locomotion in Lattices: Centipede Traversal of Obstacle‐Rich Environments

**DOI:** 10.1111/nyas.70187

**Published:** 2026-01-23

**Authors:** Christopher J. Pierce, Daniel Soto, Eva Erickson, Kelimar Diaz, Massimiliano Iaschi, Anna Lay, Daniel I. Goldman

**Affiliations:** ^1^ School of Physics Georgia Institute of Technology Atlanta Georgia USA; ^2^ School of Chemical and Biomolecular Engineering Georgia Institute of Technology Atlanta Georgia USA; ^3^ Center for Fluid Mechanics Brown University Providence Rhode Island USA; ^4^ Division of Natural Sciences Oglethorpe University Atlanta Georgia USA; ^5^ School of Mechanical Engineering Georgia Institute of Technology Atlanta Georgia USA; ^6^ School of Biology Georgia Institute of Technology Atlanta Georgia USA

**Keywords:** behavior, centipedes, gait adaptation, locomotion, myriapods, neuromechanics, terradynamics

## Abstract

Centipedes locomote through complex obstacle‐rich environments by propagating waves of body bending and limb stepping. However, little is known about how collisions with obstacles influence locomotion. In terrestrial environments such as branches or leaf litter, obstacles can both cause drag and offer affordances for the animals to generate thrust. In laboratory experiments, we challenged *Scolopendra polymorpha* (∼9 cm long, ∼1 cm wide) to negotiate model heterogeneous terrains, hexagonal and square lattices composed of thin posts. The centipedes maintained rapid motion (∼0.2 body lengths per cycle, comparable to flat ground speed) across lattices of different spacings by altering their body and limb postures in response to collisions. Several behaviors minimized deleterious limb and head collisions: the first was “prolonged limb adduction,” in which consecutive limbs fold to the body after a leading limb collides with a post, while other limbs maintained a stepping pattern. The second, occurring in narrower lattices, was “body twisting,” in which the animal propagated local body twists to locomote on its side using the posts as footholds. In some cases, the animals used a peristaltic‐like gait, previously undocumented for this species. We propose that the principles discovered here can improve morphologies and control schemes for elongate robots tasked with navigating similar terradynamic scenarios.

## Graphical abstract

We investigated how centipedes adapted their limb stepping and body bending patterns in a dense array of rigid obstacles (vertical posts). Centipedes maintained a rapid speed in the obstacle array by adapting their gaits in several ways, including adducing their limbs along their bodies to mitigate collisions, twisting their bodies into more favorable orientations, and stretching and contracting their bodies in a peristaltic motion.

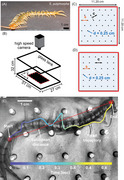

## 1 Introduction

Principles of aerodynamics and hydrodynamics have facilitated biomechanical explanations of locomotion strategies used by flying [1] and swimming [2] organisms. Despite the ubiquity of dry, cluttered habitats in the natural world, the development of a corresponding set of principles for *terradynamics* [3] is still in its infancy. This reflects the complex nature of terradynamic environments, which often feature heterogeneous, complex, nonlinear body−environment interactions and a wide variety of materials. Identifying principles of terradynamic locomotion relies in part on cataloging and describing the diverse locomotor strategies employed in these settings. The body plans and gaits (i.e., patterns of self‐deformation, including both limb‐stepping patterns and body deformation) of terradynamic locomotors display considerable variation—from limbless organisms like snakes, to bipeds, quadrupeds, hexapods, and myriapods, like centipedes—which reflects the richness and complexity of terradynamic interactions. Understanding how these different locomotors interact with their environments and how they adjust their gait can provide insights into how these organisms developed and how to build robotic systems to emulate their performance.

**FIGURE 1:**
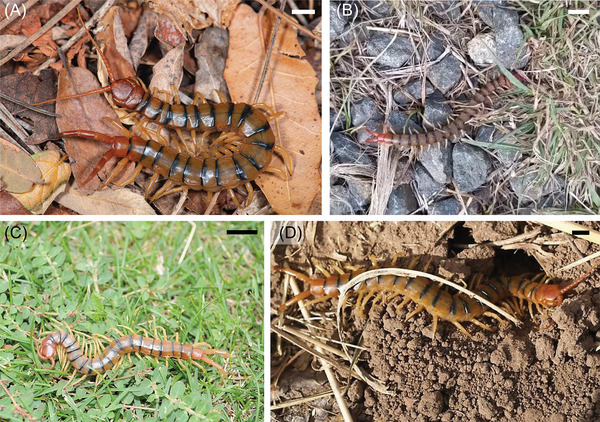
Centipedes in natural environments. *S. polymorpha* locomote across various terrains, such as (A) leaf litter (image credit: Marshal Hedin), (B) rocks (image credit: Sven Ouille), (C) plants (image credit: Richard N. Horne), and (D) holes and burrows (image credit: Margarethe Brummermann). Scale bars in A and D correspond to 1 cm. Scale bars in B and C correspond to 2 cm.

Centipedes present an interesting case study for terradynamic locomotion as they inhabit various complex environments (Figure 1) and exhibit limb‐driven locomotor modes, with and without body undulation [4, 5]. These animals locomote by propagating traveling waves of limb protraction and retraction (limb‐stepping patterns) [4, 5]. These waves are classified by the direction of propagation relative to the direction of motion. Limb aggregates (i.e., grouped limbs that make limb−substrate contact) traveling in the direction of motion (from rear to front during forward motion) are termed “direct.” Conversely, propagation of limb aggregates opposite to the direction of motion (front to rear during forward motion) is called “retrograde” [6]. Manton [5] classified various orders of centipedes based on whether they exhibited retrograde (Scolopendromorpha, Geophilomorpha, and Craterostigmorpha) or direct (Scutigeromorpha and Lithobiomorpha) limb‐stepping patterns.

Manton noted that centipedes with direct limb‐stepping patterns were unable to exhibit body undulation [5], whereas those with retrograde patterns demonstrated an increase in body wave amplitude with increased forward speed [5, 7]. Recent work has shown that different centipede species are not restricted to a single locomotive strategy, however, but can modify their gait based on their environment [8, 9, 10]. However, centipede locomotion (and legged locomotion more generally [11, 12]) in confined, obstacle‐rich settings (Figure 1D) is not well explored. In contrast, such scenarios have been extensively studied for limbless locomotors [13, 14, 15, 16, 17, 18, 19]. Given centipedes’ morphology and ability to undulate their body, it is conceivable that they would exhibit body‐driven locomotion strategies similar to those seen in limbless systems. Such work can provide insights into locomotion strategies in confined terradynamic environments and inform robot design and control for similar settings.

In this work, we present the first study of multilegged locomotion in lattices, a model heterogeneous environment, often used to study limbless systems [13, 14, 15, 16, 17, 18, 19]. We challenged *Scolopendra polymorpha*, a species known to exhibit retrograde limb‐stepping patterns and body undulation, to navigate arrays of rigid posts. We hypothesized that, at higher obstacle densities, the centipedes would forgo the use of their legs (due to leg collisions and drag induced by their sprawled posture) and leverage body‐post collisions (i.e., body‐driven locomotion), reminiscent of limbless organisms (e.g., snakes), to locomote within these obstacle‐rich environments. Instead, the centipedes adopted various strategies with continued use of their limbs (no evidence of lateral body undulation for propulsion) except in rare cases, where they exhibited a form of peristaltic body‐driven locomotion with adduced limbs. We describe these various behaviors and the conditions in which they occur. Lastly, we discuss potential insights these tests offer for understanding the interplay between body and limb locomotion in complex environments.

## 2 Methods and Materials

### 2.1 Animals

**FIGURE 2:**
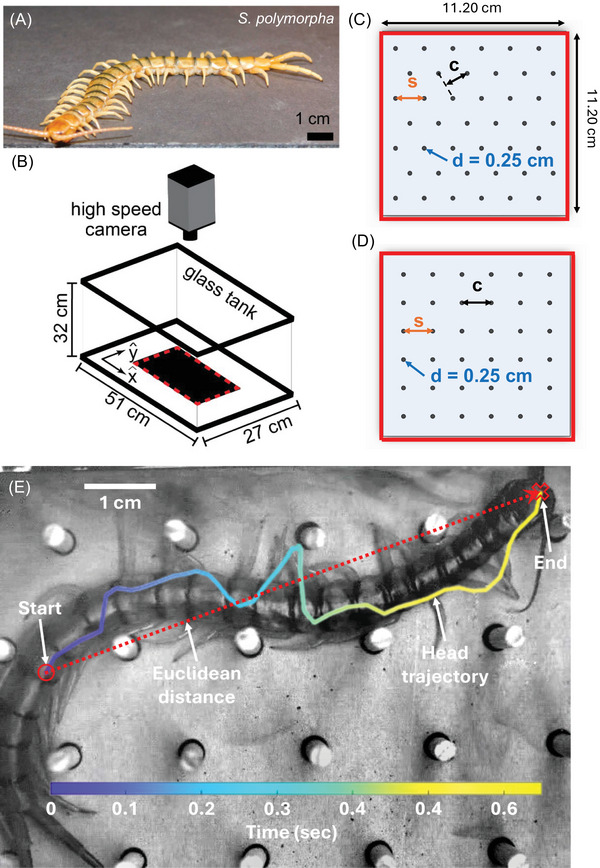
Experimental setup to study locomotion in terradynamically cluttered environments. (A) Photo of *Scolopendra polymorpha* on flat ground. Trials were performed in (B) a 51 × 27 × 32 cm^3^ glass tank with a high‐speed camera stationed above, with the tested lattice located in the red dashed box. Example hexagonal (C) and square (D) lattices with post diameter (d) and spacing (s) denoted, as well as the width of the channels (c) within the lattice. (E) Snapshot of a centipede at the end of a trial with head trajectory colored by time. The dashed red line corresponds to the Euclidean distance.

All centipedes were wild‐caught *S. polymorpha* (Figure 2A) that were obtained in Del Rio, Val Verde County, TX, USA. Nine centipedes were used in experiments, with a mean body length of 8.6 ± 1.0 cm and a mean body width of 0.8 ± 0.1 cm, with 19 body segments and leg pairs. The limbs vary in length along the body, but on average, the leg length was 1.3 ± 0.1 body widths (BW). Centipedes were housed separately in plastic containers (12 × 14 × 25 cm^3^) on a 12h:12h light:dark photoperiod (light from 6 p.m. to 6 a.m.) at room temperature (20–22°C) and were provided a source of water and fed mealworms weekly.

### 2.2 Lattice Environments

Experiments were conducted in 10 different environments placed within a glass tank (Figure 2B) with a rigid base holding 3 cm tall cylindrical wooden dowels (diameter = 0.25 cm) in either hexagonal (Figure 2C) or square (Figure 2D) lattice arrangements on a lasercut sheet of acrylic with the protective adhesive paper layer still attached. The area of each terrain was consistent (11.20 cm × 11.20 cm), while the spacing between dowels (s) was varied for testing at 1, 1.25, 1.5, 1.75, and 2 cm spacing for both configurations. Lattices were placed on a slightly raised platform (2.4 cm) within a glass tank (length = 51 cm, width = 27 cm, height = 32 cm) for each experiment (Figure 2B).

### 2.3 Kinematic Recordings

Experiments were recorded using a high‐speed camera (AOS, S‐motion) positioned directly over the lattice environments at a resolution of 1280 × 700 pixels and a frame rate of 738 frames per second. Each trial consisted of an individual centipede navigating a lattice without an external stimulus. We began a trial when the entire body was within the lattice and ended it once the head exited. For each lattice type and spacing, 5−12 trials were conducted where the centipede was completely within the terrain area for at least 0.2 s (150 frames), for an average trial duration of 0.93 ± 0.71 s. For the majority of lattice spacings, we tested between 5−7 centipedes. The only exception was hexagonal lattices with 1 cm spacing, where, due to experimental constraints (the size of our centipedes), we tested only two centipedes. The total number of trials for square and hexagonal lattices was 60 and 55 trials, respectively.

### 2.4 Image Analysis

We tracked the point on the head where both antennas meet for the first and last frames of the trial and divided that distance by the total trial duration to obtain the average Euclidean speeds (in units of body lengths per second, BL/s) of the centipedes (Figure 2E). We note that this serves as a lower bound for the instantaneous centipede speeds. We use this metric due to frequent head oscillations during tests, with no characteristic period that falls within the trial duration.

We calculated the rate of head collisions by counting the number of collisions per run and dividing by the total duration of that trial. We classified a head collision as a post coming into contact with any point on the rounded part of the centipede's head (excluding antenna).

Trials were classified as twisted or not twisted based on whether any legs were pointing toward the camera (vertically) during the run for at least 0.1 s. Completely twisted (C.T.) trials were those where half of the centipede's legs were pointing toward the camera (the other half were against the ground) throughout the trial, and partially twisted (P.T.) cases were those where an animal maintained a portion of the legs pointing toward the camera for at least 0.1 s. Additionally, since the centipedes typically followed channels within the lattice (denoted by c in Figure 2B,C), we analyzed their performance and behaviors as the function of the normalized channel width (c/BW, where BW corresponds to the body width of the centipede for that trial).

Lastly, we calculated the average leg stepping cycle duration (inverse of stride frequency) for each trial by measuring the footfall‐to‐footfall time for 2−4 limbs along the centipede's body (left and right side, front and rear). We use this average cycle duration to convert the average Euclidean speeds from body lengths per second (BL/s) to body lengths per cycle (BL/cyc), nondimensionalizing net performance to eliminate the effect of cycle variation on the speed. As noted, some trials contained bouts of prolonged limb adduction along portions of the body. We selected limbs that were actively stepping to determine the average step cycle duration for the trial.

We tracked prolonged adduction events by manually scanning through the video frame‐by‐frame and visually identifying when a limb did not perform a step for approximately the duration of one stepping cycle, based on the stepping frequency of nearby limbs. This was performed on a per‐trial basis, where we determined the fraction of total experiments under a particular lattice condition that showed any prolonged adduction of any leg, and on an individual leg basis for a single trial illustrated below in the Results and Discussion section.

## 3 Results and Discussion

### 3.1 Performance Across Lattices

**FIGURE 3:**
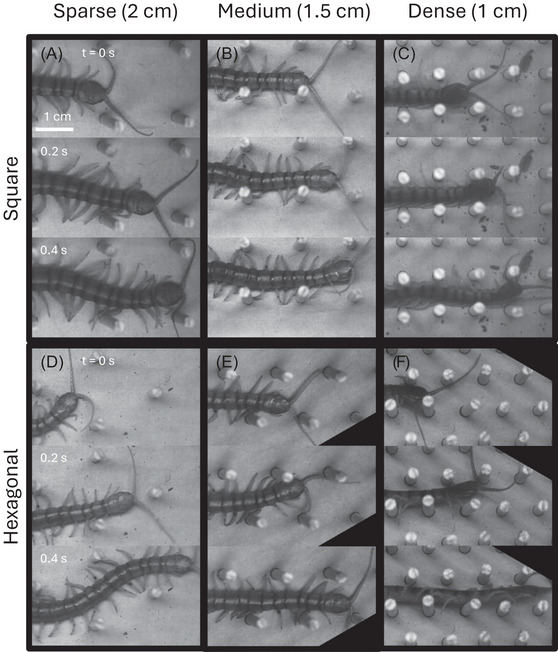
Centipede locomotion in lattices of varying density and symmetry. Image sequences of centipedes running through some of the tested square (A−C) and hexagonal (D−F) lattices. For each panel, time progresses vertically (from top to bottom). Snapshots correspond to 0.2 s intervals. Post density increases from left to right. Scale bar corresponds to 1 cm for all panels.

**FIGURE 4:**
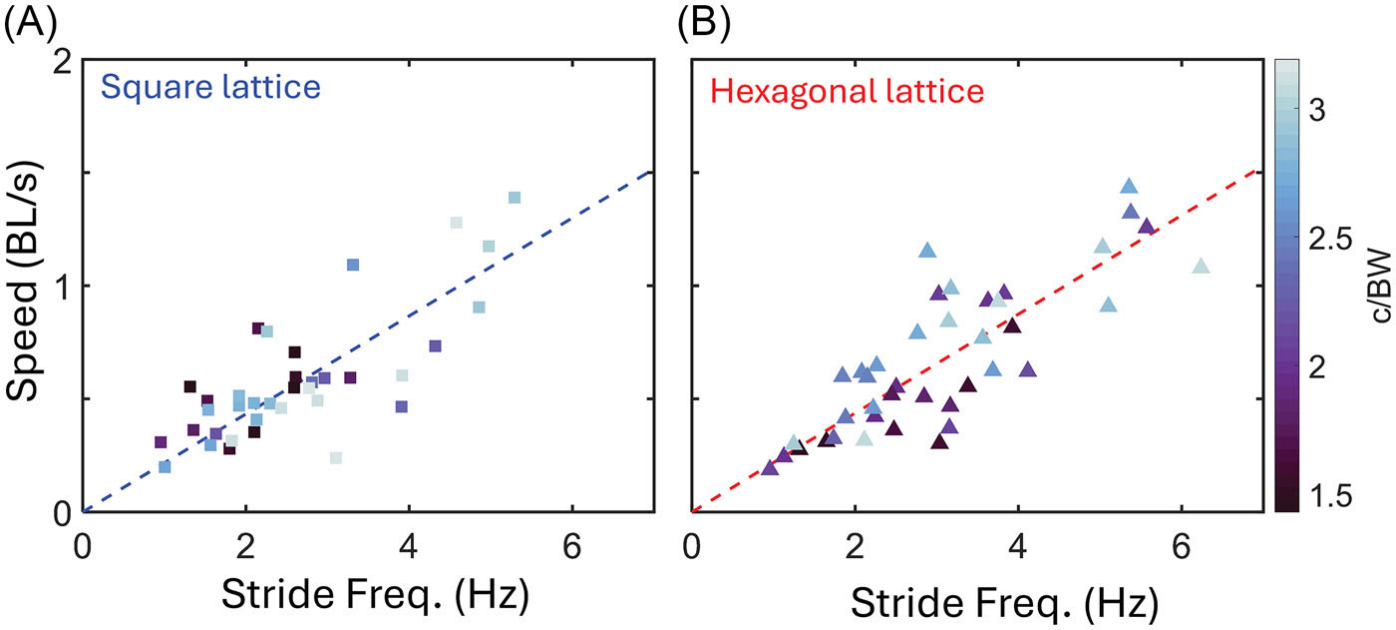
Speed versus stride frequency across lattices. Euclidean speed for (A) square and (B) hexagonal lattices. For square lattices, two to eight trials per animal (*N* = 7) were used for analysis. For hexagonal lattices, 2−11 trials per animal (*N* = 8) were used for analysis. Dashed lines are least‐squares regressions with slopes of 0.22 and 0.23 BL/s per Hz, Pearson's correlation coefficient of 0.77 and 0.82, and an *R* ^2^ of 0.58 and 0.67 for the square and hexagonal lattice, respectively.

**FIGURE 5:**
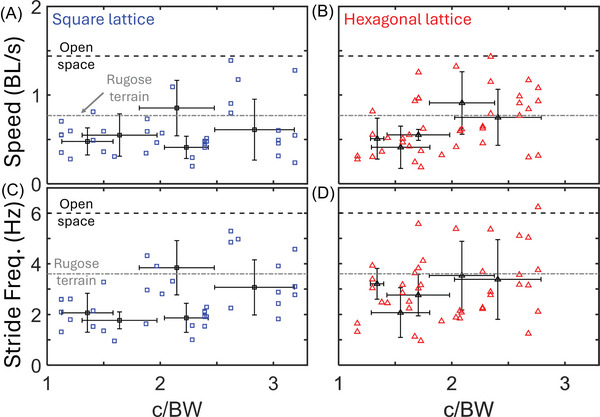
Centipede speed and stride frequency as a function of lattice spacings. Euclidean speed (A, B) in body lengths per second and stride frequency (C, D) in Hz for the square (A, C) and hexagonal (B, D) lattices versus the normalized channel width (c/BW). Dashed lines correspond to the average speed and stride frequency of the centipedes in open space (black) and terrains with rugosity of 0.44 (gray). Blue squares and red triangles correspond to individual trials in the square and hexagonal lattice, respectively. Black squares and triangles with crosses correspond to the average value per lattice spacing in the square and hexagonal lattice, respectively. Crosses represent the standard deviation for the average value (vertical line) and the average lattice spacing (horizontal line). Average lattice spacing was obtained by dividing the lattice spacing (c) by the average body width (BW) of the centipedes used for that lattice. For square lattices, 2–8 trials per animal (*N* = 7) were used for this analysis. For hexagonal lattices, 2−11 trials per animal (*N* = 8) were used for this analysis.

**FIGURE 6:**
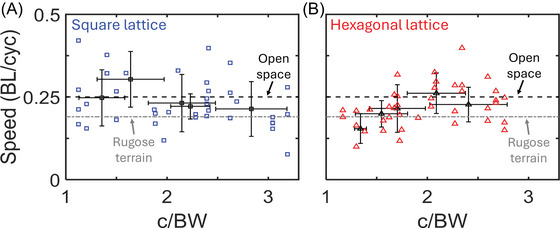
Normalized centipede speed as a function of lattice spacing. Nondimensional speed in body lengths per gait cycle (BL/cyc) for (A) square and (B) hexagonal lattices versus the normalized channel width (c/BW). Dashed lines correspond to the average speed of the centipedes in open space (black) and terrains with rugosity of 0.44 (gray). Blue squares and red triangles correspond to individual trials in the square and hexagonal lattice, respectively. Black squares and triangles with crosses correspond to the average speed per lattice spacing in the square and hexagonal lattice, respectively. Crosses represent the standard deviation for the average speed (vertical line) and the average lattice spacing (horizontal line). Average lattice spacing was obtained by dividing the lattice spacing (c) by the average body width (BW) of the centipedes used for that lattice. For square lattices, 2‐8 trials per animal (*N* = 7) were used for this analysis. For hexagonal lattices, 2−11 trials per animal (*N* = 8) were used for this analysis.

We recorded centipedes traversing hexagonal and square lattices of various densities (Figure 3) and observed a variety of behaviors. Across all lattice spacings, the speed of the centipedes was correlated with their stride frequency (Figure 4). Additionally, while the speeds for both square and hexagonal lattices were lower than the open space average (Figure 5A,B), the corresponding stride frequency (Figure 5C,D) was also less than the open space average [8], calculated for different individuals of the same species. Figure 6 shows the slopes calculated from the data presented in Figure 4, allowing us to study the effect of lattice spacing on the normalized speed (in units of body lengths per cycle, BL/cyc). We found that the centipedes maintained an approximately constant speed of 0.23 ± 0.08 BL/cyc across all square lattices (Figure 6A). In contrast, in the hexagonal lattices (Figure 6B), the centipedes showed significant changes in speed with lattice spacing. Specifically, the average normalized speed of 0.25 ± 0.06 BL/cyc when above 2 c/BW. Below 2 c/BW, the centipedes navigated the hexagonal arrays at 0.19 ± 0.06 BL/cyc, a statistically significant difference from the other spacings (*p* = 0.0075, *t*‐value = 2.8) and the square lattice (*p* = 0.08, *t*‐value = −1.8), with *p*‐values taken from a two‐sample *t*‐test. Hence, the hexagonal lattices appeared to induce changes in speed as a function of spacing, while the square lattice did not. We attribute this difference to both changes in posture (discussed in Section 4.2) and the arrangement of obstacles, each causing more turns and circuitous paths in the hexagonal case. Overall, these normalized speeds are comparable to the average speed seen in open space (0.25 ± 0.07 BL/cyc) and high rugosity terrains (0.19 ± 0.04 BL/cyc) [8], with 78% and 73% of square and hexagonal trials, respectively, falling within or above the open space average and standard deviation. These results indicate that, surprisingly, the lattices did not significantly impact the centipedes’ locomotor performance.

**FIGURE 7:**
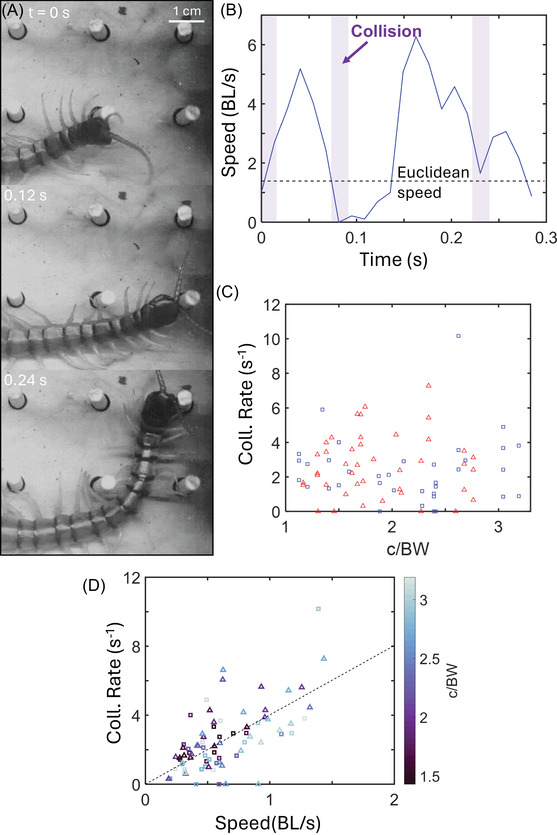
Head collisions during locomotion. (A) Image sequence of a centipede within a 1.75 cm square lattice (2.6 c/BW) colliding into posts at a rate of 10 times per second. (B) Head speed in BL/s versus time for the trial shown in (A). Shaded bars denote head‐on collisions, and the dashed line denotes the Euclidean speed (see Figure 3E). (C) Head collision rate versus lattice spacing showing a lack of correlation. (D) Head collision rate as a function of Euclidean speed for all lattices. Square and triangle markers correspond to square and hexagonal lattices, respectively. Marker color corresponds to normalized lattice width (c/BW). The dashed black line is a least‐squares regression line with a slope of four collisions per body length traveled. This trend line has a Pearson coefficient of 0.52 and an *R* ^2^ of 0.41. For square lattices, 2–8 trials per animal (*N* = 7) were used for this analysis. For hexagonal lattices, 2−11 trials per animal (*N* = 8) were used for this analysis.

One possible explanation for the unaffected performance is that the centipedes avoided deleterious collisions. However, we observed frequent head‐post collisions (Figure 7A) that resulted in rapid bending of the head away from the post, followed by near halts in the centipedes’ motion (Figure 7B) when navigating the lattices. Figure 7B shows an example where multiple head collisions occur rapidly in succession, leading to rapid fluctuations in speed. We measured the rate of collisions across all trials and found that there was no statistically significant correlation between head collision rate and lattice spacing (*p* = 0.58 and 0.92 for square and hexagonal lattices, respectively, using a Pearson Correlation test, see Figure 7C). However, we did find a positive correlation with the trial's associated speed (Figure 7D). This means that trials with higher speeds also had more collisions per second, indicating that the centipedes did not locomote faster by avoiding collisions. Instead, with decreasing lattice spacing, we observed the centipedes consistently adopt two significant changes to their posture and gait: prolonged limb adduction and body twisting. We hypothesize that these changes mitigated limb drag from the increasing obstacle densities and enabled the centipedes’ unchanging performance.

### 3.2 Posture and Gait Adaptations

**FIGURE 8:**
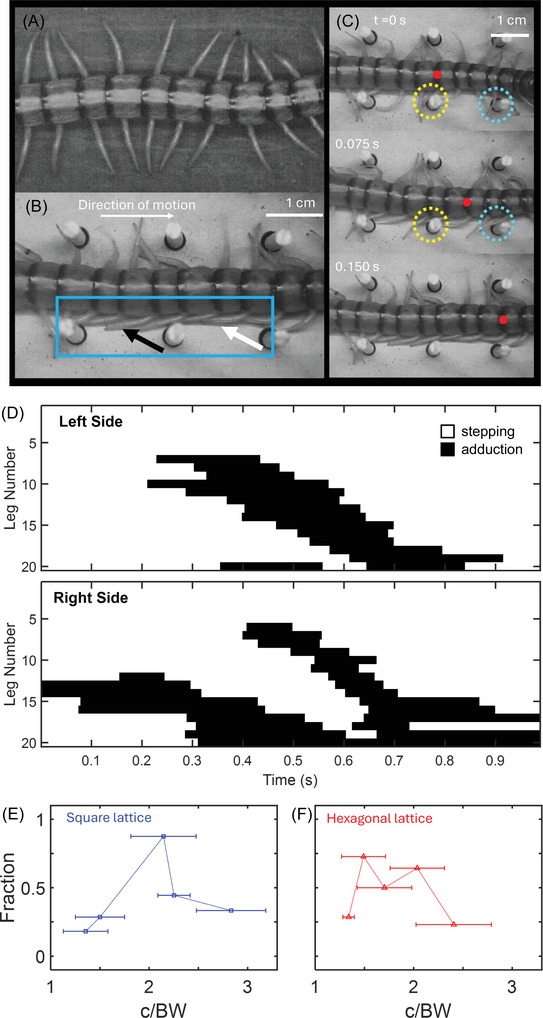
Limb adduction behaviors during lattice transit. (A) Centipede's display sprawled posture when in open space [8]. (B) Example of centipede posture during transit of a 1.75 cm square lattice (1.9 c/BW). The blue box highlights the observed limb adduction. Black and white arrows highlight limbs that are against the body when not against an obstacle. The scale bar in the upper right corresponds to panel (A) as well. (C) Image sequence across 0.15 s of centipede transitioning from sprawled to limb adducted posture after a limb's femur collides with a pillar (outlined by dashed circles). Last image in sequence (C) corresponds to image in (B). Red dot corresponds to the same body segment on the centipede, to show forward progression (from left to right) across the image sequence. (D) An example time series showing which limbs (limb 1 is the most anterior limb near the head and limb 20 is the most posterior) were adducted as a function of time (black regions) and which were stepping. Fraction of trials with prolonged limb adduction in (E) square and (F) hexagonal lattices. Horizontal mean and deviation lines correspond to the average lattice spacing and its standard deviation, respectively. The average lattice spacing was obtained by dividing the lattice spacing (c) by the average body width (BW) of the centipedes used for that lattice. For square lattices, 1–8 trials per animal (*N* = 9) were used for this analysis. For hexagonal lattices, 2−11 trials per animal (*N* = 8) were used for this analysis.

Centipedes typically have a sprawled posture where the limbs are nearly perpendicular to the body during their swing phase (Figure 8A). However, once in the lattices, the centipedes adopted a different posture (Figure 8B) where a fraction (∼20−40%) of the limbs were held against the body, pointing toward its rear, while the remaining legs continued stepping to maintain propulsion. We define this behavior as “prolonged limb adduction.” Body regions displaying prolonged limb adduction were observed even in the absence of a local mechanical constraint (i.e., while the limb was not touching a post). This is notably different than the “limb gliding” behavior noted by Diaz et al. [8], where the limbs passively bent toward the body during obstacle‐limb collisions. We observed that this adduction often occurred after a post made contact with the prefemur, femur, and/or tibia segments of a limb during its swing phase or toward the end of a stride (Figure 8C). The colliding limb then “skipped” that footfall if it was about to touch down, and subsequent legs remained adducted until the next footfall in the limb‐stepping pattern. Figure 8D shows the pattern of limbs participating in either limb stepping or prolonged adduction versus time for the square lattice example illustrated in Figure 8B,C. This trajectory illustrates a typical case, which shows the propagation of several regions of ∼3−8 legs on either side performing prolonged adduction along the body, often instigated by a collision. More extensive tracking data is currently limited by the restricted optical accessibility of legs in the lattices, which impedes automated tracking. We instead characterized the likelihood of observing this behavior across the experimental conditions.

Prolonged leg adduction behaviors were observed for all lattice spacings (Figure 8E,F), with cases above 2 c/BW primarily being when the centipedes performed turns greater than 60°. We hypothesize that this shift in posture serves to mitigate drag incurred by the limbs during forward motion by reducing the number of possible collisions with the immediate surroundings, effectively achieving a more “terradynamically streamlined” posture [11] while the legs in adjacent regions continue to protract and retract, providing a source of propulsion. In addition, since limb adduction persisted after mechanical contact and was exhibited in subsequent limbs that did not collide with the instigating pillar, this behavior is potentially an active localized response to the heterogeneous environment, similar to what was observed in *Scolopendra subspinipes mutilans* during gap traversal [20]. Taken together, our observations of limb adduction along sections of the body suggest a compromise between mitigating the drag associated with sprawled postures in cluttered terrain and providing thrust through stepping.

**FIGURE 9:**
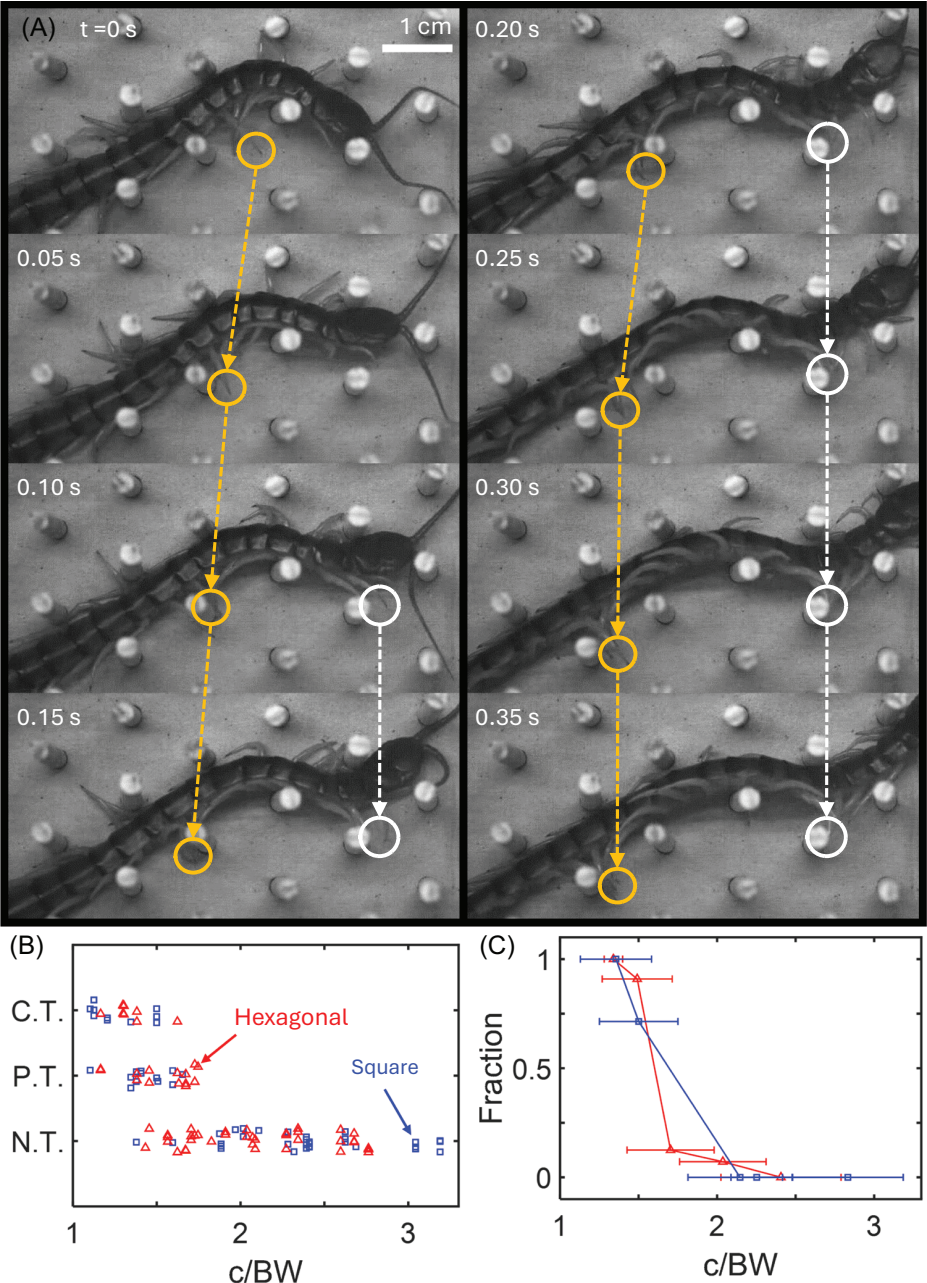
Centipedes twist to fit in narrow channels. (A) Image sequence of the onset of body twisting and locomotion in a 1.25 cm hexagonal lattice (1.9 c/BW). Orange and white arrows indicate limb aggregates traveling along the body. (B) Individual trials categorized as complete twist (C.T.), partially twisted (P.T.), and no twist (N.T.) as a function of normalized lattice channel width (c/BW). Red triangles and blue squares represent trials in hexagonal and square lattices, respectively. (C) Fraction of twisted trials (both C.T. and P.T.) versus normalized lattice channel width (c/BW). Red triangles and blue squares correspond to the hexagonal and square lattices, respectively. Horizontal mean and deviation lines correspond to the average lattice spacing and its standard deviation, respectively. Average lattice spacing was obtained by dividing the lattice spacing (c) by the average body width (BW) of the centipedes used for that lattice. For square lattices, 1–8 trials per animal (*N* = 9) were used for analysis. For hexagonal lattices, 2−11 trials per animal (*N* = 9) were used for analysis.

In high post‐density lattices (from 1 to 2 c/BW), the centipedes twisted their body and shifted from ventral−substrate contact (legs on the ground) to lateral−substrate contact (legs on the posts), using the posts for propulsion (Figure 9A). In this state, the centipedes locomoted at 0.27 ± 0.09 and 0.18 ± 0.05 BL/cyc in the dense square and hexagonal lattices, respectively, comparable to their non‐twisted performance of 0.21 ± 0.06 and 0.24 ± 0.06 BL/cyc in less dense square and hexagonal lattices, respectively. We observed that this postural shift primarily occurred when the centipedes first entered the lattice or when they underwent a turn within the lattice. This change in body−substrate contact is characterized by the centipede locally twisting its body after several limbs contact a post, presumably to favor the new foothold. Once in this twisted posture, the limb aggregates (i.e., grouped limbs that make limb–substrate contact) continued to travel along the body like in open space locomotion rather than immediately settling on posts. Further, there were several instances where limbs slipped off a post or attempted to “walk on air” (Figure 9A, yellow circle). Based on this, we hypothesize that the centipede continues its normal gait pattern when twisted. Whether the onset of twisting is active or passive is unclear; however, the centipede's body locally shifted back to an untwisted posture when its limbs slipped off a post. This suggests that, if twisting is an active response, it is only enacted for a short time before the legs reinforce this change in posture.

We classified each trial as “complete twist,” “partial twist,” or “no twist.” Completely twisted cases consisted of trials when the centipedes turned their entire body on their side, undergoing lateral−substrate contact (Figure 3F and the video in Supporting Information). Partially twisted trials consisted of at least four body segments undergoing lateral–substrate contact for at least 0.1 s (Figure 9B). Trials in which a centipede did not exhibit a complete or partial twist were classified as “no twist.” This classification revealed that transitions in body posture depended on normalized lattice width (Figure 9B,C), with twisting consistently emerging in lattices less than 1.2 c/BW. However, between 1.2 and 2 c/BW, the centipedes exhibited both twisted and untwisted postures within different trials. Notably, completely twisted cases were not observed above 1.5 c/BW (Figure 9B), with the remaining fluctuations being between “no twist” and “partial twist” postures. This variation is likely due to the different paths taken by the centipedes in the lattices (see the video in Supporting Information for selected representative examples), especially in the hexagonal trials. However, it remains unclear what drives this locomotor transition between twisted and untwisted postures between 1.2 and 2 c/BW.

**FIGURE 10:**
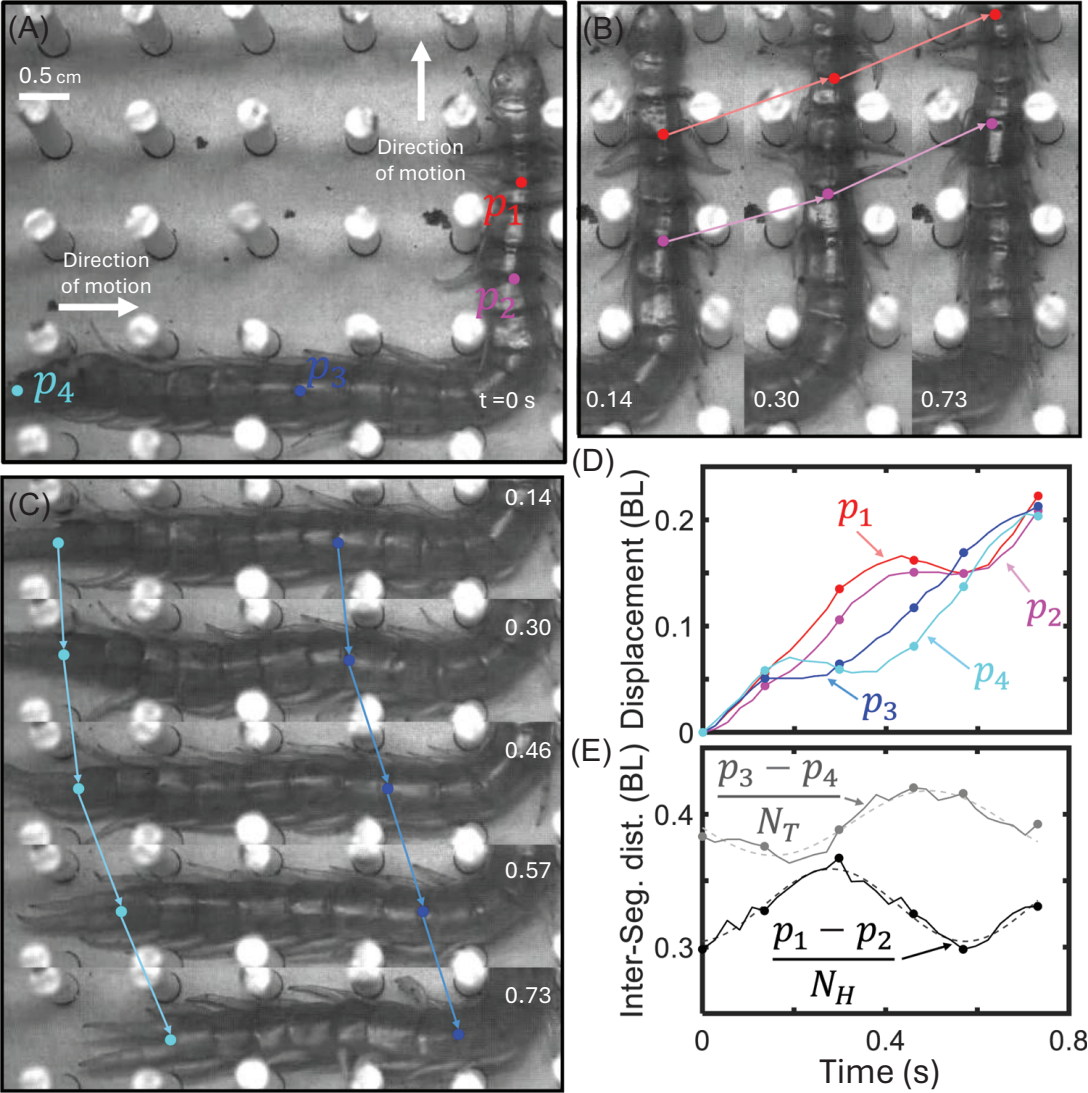
Peristaltic‐like gait used in high‐density lattices. (A−C) Image sequence of a centipede demonstrating a peristaltic‐like gait in a 1 cm square lattice (1.6 c/BW) over 0.73 s. Red, magenta, blue, and cyan dots (*p*_1_, *p*_2_, *p*_3_, and *p*_4_) are located at the intersections of centipede plates, and arrows visually indicate their displacement over the sequence. Dots were tracked every 0.027 s, and their displacement over time is shown in (D). Interpair distance (*p*_1_−*p*_2_ and *p*_3_−*p*_4_), normalized by the number of segments between them (*N_H_* = 3 and *N_T_* = 7, respectively), versus time shows the extension and contraction of the front (solid black line) and back (solid gray line) portions of the centipede's body. Dashed lines correspond to a sine fit to each intersegment distance.

Alongside twisting, the centipedes made use of the longitudinal compressibility of their bodies for different effects within the lattices. In some trials (13 out of 115) and across the full range of tested lattice spacing (1.1−3.2 c/BW), the centipedes compressed multiple body segments to rapidly reverse or reorient their head within the lattice without moving the back half of their body (see Supporting Information). Additionally, in lattices between 1.1 and 1.7 c/BW, the centipedes randomly (7 of 115 trials) performed a peristaltic‐like gait where they used the expansion and contraction of their body segments to move forward instead of performing strides with their limbs. An example trial is shown in Figures 10A–C, where the centipede underwent a 90° turn and proceeded to periodically stretch and contract its body segments in its front (Figure 10B) and back (Figure 10C) halves. This peristaltic‐like gait is similar to that of *S. polymorpha* [21] and Geophilomorpha [4] burrowing gaits and seems to occur when the centipedes are forced to flee through confined scenarios with limited limb mobility. To quantify the magnitude of body stretching and compression during peristalsis, we tracked four points along the centipede's midline (*p*_1_, *p*_2_, *p*_3_, and *p*_4_) over time (Figure 10D).

We found that the distance between the anterior (*p*_1_ and *p*_2_) and posterior (*p*_3_ and *p*_4_) points oscillated with similar frequencies (1.71 ± 0.07 and 1.47 ± 0.09 Hz, respectively) and amplitudes (0.027 ± 0.003 and 0.024 ± 0.004 BL, respectively) with a phase offset of 0.49 ± 0.11 radians (Figure 10E). Future work will be done to identify whether this peristaltic‐like gait is a traveling wave similar to the limb‐stepping and body undulation waves, and under what specific conditions this gait occurs. Regardless, these relatively infrequent body compression behaviors are noteworthy as they demonstrate the centipedes’ locomotor versatility and enable locomotion in complex scenarios where the other behaviors noted above are insufficient or lacking due to limited limb mobility.

## 4 Conclusion

We studied the dynamics of a multilegged locomotor, the centipede *S*. *polymorpha*, in lattices, a model obstacle‐rich terradynamic environment often used to study limbless systems [13, 14, 15, 16, 17, 18, 19]. We discovered that, instead of using their bodies in these confined settings (similar to limbless undulators like snakes and nematode worms), the centipedes continued to use their limbs as their main source of propulsion and experienced little to no change in their net displacement per cycle. This occurred despite frequent head collisions that caused the centipedes to pause or redirect. We hypothesize that the centipedes leveraged their versatile morphology and changed their posture and gait during each trial such that the obstacles and collisions did not affect their net performance. We describe two distinct behaviors that we posit are crucial for the centipedes’ success in the lattice: prolonged limb adduction and body twisting. The first has not been noted before to the best of our knowledge, while the latter is similar to a behavior noted by Manton [7], where *Scolopendra* turns on its side to pass through confined spaces such as between blades of grass or bamboo. In this work, we show under what conditions these behaviors typically occur and note a rare peristaltic‐like gait that we posit serves as a contingency maneuver for when limb adduction and body twisting are insufficient for the complexity of the centipede's path and local environment. Further insights into the control principles governing these behaviors should enhance elongate legged robots [22, 23, 24, 25, 26, 27, 28] that must maneuver in terradynamically complex environments, like those in search and rescue settings [29] and agriculture.

## Author Contributions

Conceptualization: C.J.P., D.S., and D.I.G. Methodology: C.J.P., D.S., K.D., and D.I.G. Animal experiments: E.E. and K.D. Data curation: E.E. and K.D. Analysis: E.E., K.D., M.I., and A.L. Formal analysis: C.J.P., D.S., and D.I.G. Visualization: C.J.P., D.S., and D.I.G. Writing: C.J.P., D.S., and D.I.G. Supervision: D.I.G.

## Conflicts of Interest

The authors declare they have no conflicts of interest.

## Supporting information




**Supporting Information**: Legged locomotion in lattices SI Final_SD

## Data Availability

All videos and spreadsheets are available upon request.
